# ASGR1 deficiency diverts lipids toward adipose tissue but results in liver damage during obesity

**DOI:** 10.1186/s12933-023-02099-6

**Published:** 2024-01-28

**Authors:** Monika Svecla, Lorenzo Da Dalt, Annalisa Moregola, Jasmine Nour, Andrea Baragetti, Patrizia Uboldi, Elena Donetti, Lorenzo Arnaboldi, Giangiacomo Beretta, Fabrizia Bonacina, Giuseppe Danilo Norata

**Affiliations:** 1 Department of Pharmacological and Biomolecular Science “Rodolfo Paoletti”, Università degli Studi di Milano, Milan, Italy; 2 Department of Biomedical Science for Health, Università degli Studi di Milano, Milan, Italy; 3 Department of Environmental Science and Policy, Università degli Studi di Milano, Milan, Italy

**Keywords:** ASGR1, Obesity, Liver, Lipoprotein metabolism, Adipose tissue

## Abstract

**Background:**

Asialoglycoprotein receptor 1 (ASGR1), primarily expressed on hepatocytes, promotes the clearance and the degradation of glycoproteins, including lipoproteins, from the circulation. In humans, loss-of-function variants of ASGR1 are associated with a favorable metabolic profile and reduced incidence of cardiovascular diseases. The molecular mechanisms by which ASGR1 could affect the onset of metabolic syndrome and obesity are unclear. Therefore, here we investigated the contribution of ASGR1 in the development of metabolic syndrome and obesity.

**Methods:**

ASGR1 deficient mice (*ASGR1*^−/−^) were subjected to a high-fat diet (45% Kcal from fat) for 20 weeks. The systemic metabolic profile, hepatic and visceral adipose tissue were characterized for metabolic and structural alterations, as well as for immune cells infiltration.

**Results:**

*ASGR1*^−/−^ mice present a hypertrophic adipose tissue with 41% increase in fat accumulation in visceral adipose tissue (VAT), alongside with alteration in lipid metabolic pathways. Intriguingly, *ASGR1*^−/−^ mice exhibit a comparable response to an acute glucose and insulin challenge in circulation, coupled with notably decreased in circulating cholesterol levels. Although the liver of *ASGR1*^−/−^ have similar lipid accumulation to the WT mice, they present elevated levels of liver inflammation and a decrease in mitochondrial function.

**Conclusion:**

ASGR1 deficiency impacts energetic homeostasis during obesity leading to improved plasma lipid levels but increased VAT lipid accumulation and liver damage.

**Supplementary Information:**

The online version contains supplementary material available at 10.1186/s12933-023-02099-6.

## Introduction

Excessive adipose tissue accumulation leads to metabolic syndrome and obesity, which are among the primary contributors to the increased morbidity of cardiovascular disease, insulin resistance, and type 2 diabetes [1]. Common features of these manifestations include altered lipid and glucose levels in the circulation, along with chronic inflammation. To maintain the lipid metabolic homeostasis, the primary physiological response is to store the excess of calories in the form of triglycerides (TGs) in the adipose tissue, despite the very limited activity in synthesis, catabolism, and efflux of cholesterol in this tissue [2]. Consequently, TGs are taken up by adipose tissue embedded in triglyceride-rich lipoproteins, mainly as very low-density lipoproteins (VLDL) and their remnants, after the hydrolysis within the adipocytes; once in the cells, they are packed within lipid droplets and available for energetic purposes, following lipolysis [3]. Furthermore, adipose tissue can also store cholesterol, accounting for approximately 20% of the total intracellular cholesterol pool, a percentage that can even reach up to 50% in obesity [4], which is taken up mainly from high-density lipoproteins (HDL) [5].

In contrast, the liver is a metabolically active tissue and plays a central role in the synthesis, catabolism and excretion of lipids and lipoproteins. Indeed, during prolonged consumption of a high-fat diet (HFD), the liver increases the lipid uptake as well as the capacity to secrete triglycerides and cholesterol-rich lipoproteins, in the form of VLDLs which in circulation are converted into low-density lipoproteins (LDLs) [6, 7]. Only a fraction of the excessive fat intake contributes to the increased lipoprotein synthesis while the rest accumulates within the hepatocytes, leading to increased ectopic lipid storage. Over time, this can result in cellular damage, affecting the endoplasmic reticulum (ER) and mitochondria [8], which promote the production of free reactive species, and in turn, can contribute to chronic liver inflammation and, eventually, liver failure [9].

Liver lipid metabolism is regulated by receptors recognizing lipoproteins. In addition to the homologous and non-homologous members of the LDL receptor (LDLR), also the asialoglycoprotein receptor 1 (ASGR1), the functional subunit of asialoglycoprotein receptor ASGPR [10], is involved in the clearance of LDLs and chylomicron remnants from the circulation [11–13]. ASGR1 is a glycan-binding receptor, which primarily mediates the uptake and the removal of circulating desialylated glycoproteins, in which sialic acid, as terminal glycan moiety is removed, and galactose or N-acetylgalactosamine is exposed [14, 15]. Recent genetic studies indicated that a rare loss-of-function variant in the ASGR1, resulted in a significant reduction in blood levels of “non-HDL” cholesterol, and a 34% reduction in cardiovascular diseases  risk [16]. The entity of this reduction was far distant from the one determined by the more common atheroprotective loss-of-function variants, as in the LDLR, Apolipoprotein B (APOB) and Proprotein Convertase Subtilisin/Kexin Type 9** (**PCSK9) genes, implying that the benefit could be attributed to other non-redundant effects resulting from ASGR1 reduction.

At the molecular level, it was shown that ASGR1 deficient mice, present an increased activation of the Liver X Receptor (LXR), which promotes cholesterol efflux and excretion into the bile, thus contributing to the reduction of circulating lipid levels [17]. It is expected, however, that increased LXR activation in the liver results in an increased synthesis of fatty acids, via the activation of sterol regulatory element-binding protein (SREBP)*-*1c [18]. However, ASGR1 deficient mice, in the same study showed additional metabolic changes related to increased activation of AMP-activated protein kinase (AMPK), which plays a role in suppressing hepatic fatty acid synthesis by inhibiting SREBP-1c, thus preventing the adverse effects of steatosis [19].

Under obesogenic conditions, such as high-fat diet feeding, it is unclear whether ASGR1 can potentially reprogram hepatocyte metabolism and have a favorable effect on lipid profiles, thereby impacting the onset of metabolic syndrome and obesity. To this aim, we fed *ASGR1*^−/−^ mice with a high-fat diet (45% Kcal from fat) for 20 weeks and observed that *ASGR1*^−/−^ mice present a hypertrophic adipose tissue alongside alteration in lipid pathways. Intriguingly, glucose levels following GTT or ITT tests were similar in *ASGR1*^−/−^ mice maintain a comparable response to an acute glucose and insulin challenge in circulation compared to WT, coupled with a notable decrease in circulating lipid levels. These data point to ASGR1 as a checkpoint controlling lipid accumulation among different tissues involved in their metabolism.

## Material and methods

### Animals

Wild type and *ASGR1*^*−/−*^ mice (B6.129S4‐Asgr1tm1Sau/SaubJxm) were purchased from Jackson Laboratories (Bar Harbor, Me, USA). The mice were kept under controlled light/dark cycle (12 h of light/12 h of dark) and temperature-controlled conditions (21°C). Starting at 8 weeks of age, for 20 weeks, mice were fed on high fat diet (HFD- 45% Kcal from fat, Research Diets, Inc Cat#D12451.) and water, provided ad libitum. Body weight and food intake were measured weekly. All the described experiments were performed conformed to the guidelines from 2010/63/EU directive of the European Parliament and were approved by Ethical Committee of the University of Milan and Italian Ministry of Health (Progetto di Ricerca 91/2020).

### Glucose homeostasis

The glucose tolerance test (GTT) and insulin tolerance test (ITT) were determined as previously described [20]. In brief, 14-h fasted mice were intraperitoneally injected with glucose (1 gr/kg body weight) for GTT, while 4-h fasted mice were intraperitoneally injected with insulin (0.75 IU/kg body weight, Humulin R, Lilly) for ITT. Blood glucose levels were tested before and at 20, 40, 60, 90, and 120 min after glucose or insulin injection by tail bleeding.

### Metabolic analysis

Metabolic analyses were measured by indirect calorimetry utilizing a computer-controlled system (Promethion Metabolic Screening, Sable Systems International, Las Vegas, NV). All mice had ad libitum access to food and water. The structure of the metabolic cages provides for the presence of detectors for mice, food, and water weight and gases analysis as described previously [21]. Data were collected for 48 h consecutively, after acclimatation of animals to the metabolic cages for 48 h. Respiratory gases were measured with an integrated fuel cell oxygen analyzer, a CO_2_ spectrophotometric analyzer and a capacitive partial pressure water vapor analyzer (GA3m1, Sable Systems International). In operating mode, the Promethion system employs a negative pressure system. The multi-channel mass flow generator (FR8-1, Sable Systems International) measures and controls airflow. The current flow rate was set at 2000 ml/min. Oxygen consumption (O_2_) and carbon dioxide (CO_2_) production were measured for each mouse for 1 min at 7-min intervals. The data is then evaluated using the web application for Indirect Calorimetry Analysis (https://calrapp.org/).

### Adipose tissue and liver proteomics

Visceral adipose tissue (VAT, gonadal depots) from the WT and *ASGR1*^*−/−*^ mice (n = 6/group) were pooled up to 100 mg and processed as previously described, with minor modifications [22]. In brief, fat pads were sonicated for 30s twice with breaks on ice (30s/30s), with buffer consisting of 50 mM HEPES, 1% Triton, 100 mM NaF, 10 mM Na-orthovanadate, 10 mM EDTA, 0.2% SDS, 100 mM NaCl. Homogenates were lysed for 5 min on ice with Tissue Ruptor (Qiagen, Venlo, Netherlands). Proteins were separated from fat and debris by centrifugation at 20,000 × *g* and 4 °C for 30 min. Thereafter, proteins were precipitated using four times the volume of acetone per sample and incubated for 15 min at – 80 °C and 120 min at −20 °C. Proteins were pelleted by centrifugation at 16,000 × *g* and 4 °C for 15 min, then air-dried and resuspended in buffered 8M urea, Tris–HCl 0.1 M, pH 8.5 supplemented with protease inhibitors at a ratio of 1:100 (Cell Signaling, Cat# 5872S). For the liver proteomics analysis we proceeded as previously described [23]. Briefly, liver from each genotype (n = 3/group) were pooled up to 20 mg and lysed with urea 8M, Tris–HCl 0.1 M pH 8.5 in the presence of protease inhibitors at a ratio of 1:100 (Cell Signaling, Cat# 5872S) for 60 min at 4 °C with constant shaking. Next, both VAT and liver samples were centrifuged for 30 min at 14,000*g* at 4 °C. The supernatant containing the proteins extracted was collected and quantified by Lowry protein assay. A volume corresponding to 50 μg of VAT proteins and 10 μg of liver proteins were then dried completely using a vacuum concentrator at 45 °C for 45 min and later resuspended in 10 μl of water with the addition of 10 μl of ammonium bicarbonate solution 50 mM (final pH 8.5). Proteins were reduced following incubation with DTT (final concentration 5 mM), for 30 min at 55 °C. Protein alkylation was then performed at RT, by incubating with iodoacetamide (final concentration 15 mM), for 20 min in the dark. Trypsin digestion (enzyme to protein ratio of 1:20), was performed overnight at 37 °C, and terminated by acidification with trifluoroacetic acid (final percentage: 1%). LC–MS/MS analysis were carried out in a Dionex Ultimate 3000 nano-LC system (Sunnyvale CA, USA). The system was connected to an orbitrap Fusion™ Tribrid™ Mass Spectrometer (Thermo Scientific, Bremen, Germany) equipped with a nanoelectrospray ion source working in positive ion mode. The peptide mixtures present in the samples were initially concentrated onto an Acclaim PepMap C18 column (particle size 5 µm, 100 µm x 2cm, Thermo Fisher Scientific) and separated at 35°C on an EASY-Spray PepMap RSLC C18 column (particle size 3 µm, 75 µm × 25 cm, Thermo Fisher Scientific). The separation employed mobile phase A (0.1% aqueous formic acid) and mobile phase B (0.1% aqueous formic acid /acetonitrile (2:8)). The flow rate was maintained at 300 nL/min. MS spectra were collected in positive ion mode at a resolution of 120.000 (m/z range of 375–1500 Da), employing in the data-dependent mode, with a cycle time of 3 s between master scans. Fragmentation was induced by higher energy collisional dissociation (HCD), utilizing a collision energy level of 35 eV.

### Proteomics data processing and analysis

The raw data files were converted to centroid format (mzML format) using the MSconvert tool of the software ProteoWizard (version 3.0.1957) [24]. The mzML files were then analyzed using an OpenMS nodes operating within the open-source software platform KNIME® (version 4.6.) [25]. Peptides were identified by combining the search engines described previously [26], against the Uniprot FASTA database for mouse (Jan 2022, 17.527 entries), and a common contaminant proteins database while the spectral library required by the SpectraST search engine was downloaded from the website www.peptideatlas.org (file NIST_mouse_IT_2012-04-21_7AA.splib). Fragment mass tolerance was set at 0.02 Da and precursor mass tolerance at 5.0 ppm. Peptide sequences were indexed through the OpenMS PeptideIndexer node, setting leucine/isoleucine equivalence. Protein inference was then carried out using the Protein Inference Analysis (PIA) algorithm using the default parameters [27]. Protein abundance estimates were calculated with prior generation of spectral feature by the node FeatureFinderMultiplex followed by PIA-assisted FDR-multiple scores estimation and filtering (combined FDR score < 0.01), their ID mapping and combination with peptide IDs, their subsequent alignment, grouping and normalization (e.g., MapAlignerIdentification, FeatureUnlabeledQT and ConsensusmapNormalizer nodes). Protein and peptide label free quantification (LFQ) was then computed with the OpenMS ProteinQuantifier node based on intensities of the n = 3 most abundant identified peptides. The corresponding output files were read as tables of the CSV reader node output and exported into Microsoft Office Excel for further formatting and statistical elaboration. The abundances were Log_2_ transformed and the proteins with at least three replicates were taken into consideration for statistical analysis. For proteome downstream analysis, Gene Ontology (GO) using the Database for Annotation, Visualization, and Integrated Discovery platform (DAVID, NIAID, Bethesda, MD) [28] and Ingenuity Pathway Analysis (IPA®, QIAGEN, Redwood City, CA) [29] were used.

### Lipid profile

Blood samples were collected through intracardiac puncture and plasma was separated by centrifugation (8,000 rpm for 10min). Total plasma cholesterol and triglycerides were measured from frozen plasma by standard enzymatic techniques using the Cholesterol CP KIT (ABX Pentra, HORIBA Medical) or the triglyceride CP KIT (ABX Pentra, HORIBA Medical). Cholesterol and triglyceride concentrations were read by spectrophotometer at 490 nm (Bio-Rad iMark microplate reader). Folch extraction was used for the lipid isolation from livers as previously described [30]. In brief, a chloroform/methanol (2:1) solution in 20-fold excess was added to the tissue lysates, which were then left in agitation for 2 h at room temperature. After 15min centrifugation at 3200 × *g*, 0.2 volume parts of PBS were added to the supernatant. Samples were vortexed and centrifuged 800 × *g* for 15 min. The lower phase was collected and dried in nitrogen stream. One hundred μl of 2% Triton X-100 in chloroform were added and dried under nitrogen gas. Thereafter, the samples were dissolved in 100 μl H_2_O and lipid concentrations were measured.

### Lipid extraction and fatty acid content

Visceral adipose tissue (n = 6/group) have been extracted three times using CHCl_3_/CH_3_OH (2:1), KCl 0.05% and butylated hydroxytoluene as antioxidant. Internal standards (stigmasterol, cholesteryl heptadeacanoate, triheptadecanoin, and nonadecanoic acid) were also added to the extraction mixture to measure the mass of the lipid subclassese (free-, esterified cholesterol, triglycerides, and free fatty acids). The total fatty acid profile analysis (qualitative analysis) has been directly performed on the aliquots of the lipid extract, lipid subclasses (free- and esterified cholesterol, triglycerides, free fatty acids) have been separated by thin layer chromatography (TLC) silica gel 60 plates (20X20 cm with concentrating zone 2.5 cm; Merck). After development (eluent hexane/diethylether/acetic acid 80:20:1) and dichlorofluorescein staining, lipid subclasses have been identified by comparison of their spots with those of proper standards. Later, aliquots of whole lipid extract or silica gel containing the lipid subclasses were derivatized by methanolic HCl 3N for 120 min at 80 °C. The obtained methylated fatty acids have been extracted by hexane/water and detected by gas–liquid chromatography (GLC DANI 1000, DANI Milano) equipped with a flame-ionization detector, hydrogen as gas carrier, and a HTA autosampler (HTA instruments, Brescia). For cholesteryl esters, triglycerides and free fatty acid analysis a MEGA-5 capillary column (0.25 mm internal diameter, 30 m length; MEGA Columns, Legnano) is used and the oven temperature programmed as follows: 150–190 °C at 8 °C/minute; from 190 to 210 at 4 °C/minute and to 320 °C at12 °C/minute, hold for 6 min, for a total run of 25.5 min. The identification of each fatty acid has been achieved by comparing the retention times of each peak with those of a standardized mixture of methylated fatty acids (FAME MIX 37; Merck). The relative amount of each fatty acid has been calculated by the Clarity Software, (Clarity, Prague, Czech Republic), by summing the areas under the curve of each peak and calculating the relative percentage. Free cholesterol has been scraped out of the TLC, resuspended in hexane/isopropanol, and analyzed without derivatization by using a DB-5 (0.32 mm internal diameter, 30 m length) column (J&W; Service TL, Milano). Temperature has been raised from 220 °C up to 310 °C, at 20 °C/minute and left for additional 5 min. Fatty acids have been categorized into saturated-, monounsaturated and polyunsaturated by summing their relative %, the mass of each lipid subclass has been calculated by comparison with the AUC of the corresponding internal standard and normalized by the total protein content determined by the bicinchoninic assay. Results are expressed as ug lipid/ug proteins.

### Phospholipid quantitative analysis

Aliquots of the lipid extract have been analyzed for their phospholipid content by putting them in clean glass tubes together with a solution of sulfuric acid (1 N). After an overnight incubation at 150  C, hydrogen peroxide (30%) has been added and tubes re-incubated at 150 C until the dark color disappeared. Ascorbic acid (0.1 g/ml), together with water and ammonium molibdate (0.01 g/ml in H_2_SO_4_) has been added and the solutions incubated for further 20 min at 55  C. The amount of phosphorus in phospholipids has been estimated by reading the absorbances of the samples by a spectrophotometer at a 750 nm wavelength and by comparing them with the values obtained by a proper standard scale.

### Oral lipid tolerance test and lipoprotein production test

After an overnight fast, the basal triglycerides and cholesterol were determined in the blood obtained from the caudal vein after a 15 min application of local anesthetic (Lidocaine 2.5% and Prilocaine 2.5%). After that, for the oral lipid tolerance test an oral bolus of 350 μl oil (EVO oil) was administered and, for the lipoprotein production test an intraperitoneal injection of poloxamer 407 (500 mg/kg, Merk Cat. #16758) was administered. Following the bolus and intraperitoneal injection, triglyceride levels were measured by taking a drop of blood from the caudal vein at 1, 2 and 4 h after the oral bolus or poloxamer injection.

### Fast protein liquid chromatography of plasma lipoproteins

Total cholesterol and triglyceride content in the main lipoprotein classes (VLDL, LDL and HDL) was determined using fast protein liquid chromatography (FPLC). The lipoproteins were separated with Superose 6 column (GE Healthcare, Chicago, IL, USA) coupled with NGCTM chromatography system FPLC (BioRad laboratories Inc., Hercules, CA, USA). As an eluent solution 0.15M NaCl with 0.01% (w/v) EDTA and 0.02% (w/v) NaN_3_ (pH 7.4). The plasma volume injected was 0.3 ml and from each eluted fraction 0.5 ml is collected and cholesterol and triglycerides concentration are measured with the above-mentioned kit.

### Alanine Aminotransferase (ALT) and Aspartate Aminotransferase (AST) measurments in plasma

Plasma levels of Alanine Aminotransferase (ALT) and Aspartate Aminotransferase (AST) were measured with commercially available kits run on automatic analyzer (Randox, Crumlin, Ireland), following the method indicated International Federation of Clinical Chemistry (IFCC) (for ALT: cat#AL8006; R1 6 × 56 ml (L) and R2 6 × 20 ml (Mod. IFCC); for AST: Cat#AS8306; R1 4 × 20 ml (L) and R2 4 × 7 ml).

### Sample preparation for immunophenotyping

Fresh visceral adipose tissue (VAT) was placed on ice in a 6-well plate and cut into small pieces in 2 ml of PBS 5% bovine serum albumin solution. After the addition of collagenase (200 mg/mL final concentration, NB4 standard grade) and CaCl_2_ (5 mM final concentration) samples were incubated at 37 °C for 40 min under agitation. Samples were then topped up to 10 ml with MACS buffer (containing 1×PBS, 2% FCS, 2 mM EDTA) and filtered on a sterile gauze and subsequently on a 100 μm and 70 μm cells strainers [31]. Following this, the cells were washed and resuspended in the antibodies mix for 30 min at 4 °C.

For liver processing, 800 to 1000 mg of liver per sample was shattered on a 100 μm cell strainer and adjusted to a volume of 10 ml with MACS buffer. Afterwards, the lysate was centrifuged at 620 rpm for 1 min, break-off, and the supernatant was transferred to a 50 ml tube. Subsequently, the transferred supernatant was centrifuged for 8min at 1720 rpm, on high brake. The resulting pellet was resuspended in 7 ml of 37.5% Percoll™ plus solution (Cytiva, Uppsala, Sweeden) containing 37.5% Percoll™ plus solution, 3.75% PBS10× and 58.75% RPMI1640 (Euroclone, Milan, Italy). Next, the resuspended pellet was transferred into a 15 ml tube and centrifuged at 2270 rpm for 30 min break-off. The obtained pellet after the centrifugation was lysed with 1 ml of 1 × red blood cell lysis buffer (Invitrogen, Thermo Fisher Scientific). Following this step, the cells were washed and resuspended in the antibodies mixture for 30 min at 4 °C. Samples were acquired with BD LSRFortessa^TM^ X-20 Cell Analyzer (BD Bioscience). Antibodies used are listed in Additional file 2: Table S1 and the gating strategies are listed in the Additional file 1: Figs. S4 and S5.

### Quantitative real-time PCR (qRT-PCR)

Total RNA was isolated from VAT (n = 6/group) is homogenized with Tissue Lyser II (Qiagen) and total RNA was extracted using RNeasy Lipid tissue mini kit (Qiagen). RNA was assessed for quality and quantity using absorption measurements (NanoDrop 1000 Spectrophotometer, Thermo Fisher Scientific) and transcripted in cDNA with iScript Reverse Transcription Supermix for RT-qPCR (Bio-Rad). The same amount of RNA was retrotranscribed using iScript™ cDNA synthesis kit (BioRad). Gene expression analysis was performed using SYBR Green Supermix (Thermo Fisher Scientific) in CFX connect light cycler (BioRad, Cat#1708841). The thermal cycling profile was a two-step amplification process (95 °C for 5 min, followed by 45 cycles of 95 °C for 10 s and 55 °C for 30 s) and the sequences of the qPCR primers are listed in Additional file 2: Table S4**.**

### Histology

Parts of VAT, SCAT, and liver were fixed in 4% paraformaldehyde (Sigma-Aldrich) overnight at 4 °C, dehydrated and embedded in paraffin as previously described [32]. Tissue Sections (5 μm) were prepared by using a microtome and stained with hematoxylin and eosin (Sigma-Aldrich). VAT and SCAT were acquired using the Axiovision Zeiss software (10 × magnification). The quantification of adipocytes area was performed using ImageJ software with the Adiposoft plugin [33]. For the liver section, whole slices were acquired with the Nanozoomer S60 slide scanner (Hamamatsu, Tokyo, Japan). The images were then magnified and saved at 20 × with the NDPview2 software (Hamamatsu) to show only the considered area, which was then quantified with ImageJ software. Area covered by small lipid droplets within hepatocytes was evaluated as microsteatosis, whereas area covered by large lipid droplets disrupting the cell morphology (small nuclei pushed to the cell border) was quantified as macrosteatosis [34]. Inflammatory foci were evaluated as groups of > 5 close non-hepatocytic nuclei [35].

### Transmission electron microscopy (TEM)

Liver fragment from each genotype (n = 3/group) was obtained and fixed in 3% glutaraldehyde (Acros Organics, Thermo Fisher Scientific, Waltham, MA, USA) diluted in 0.1 M Sorensen phosphate buffer (pH 7.4) at 4 °C overnight. Subsequently, the livers were post-fixed with 1% osmium tetroxide in 0.1 M Sorensen phosphate buffer for 30 min, dehydrated, and araldite embedded (Fluka, Sigma Aldrich, St. Louis, MO, USA). Ultrathin sections were obtained with an Ultra-cut ultramicrotome (Reichert-Jung, Leica, Microsystems GmbH, Wetzlar, Germany), stained with uranyl acetate and lead citrate, and observed with Talos 120 (Thermo Fisher Scientific). The TEM images were acquired at 6700X magnification in randomly selected fields. Results are expressed as diameter (µm) and number of mitochondria per area for each analyzed section and were quantified by ImageJ software [36].

### Statistics

Data are expressed as the mean per group ± SEM and analyzed in accordance with the figure legends. For this study *p < 0.05, **p < 0.01 and ***p < 0.001 were considered significant. Comparisons within groups were made by using T-test for unpaired samples and for multiple comparisons, the ANOVA test was applied. For GO, the pathways with FDR < 0.05 were taken into consideration.

## Results

### ASGR1 deficiency promotes white adipose tissue hypertrophy.

**Fig. 1:**
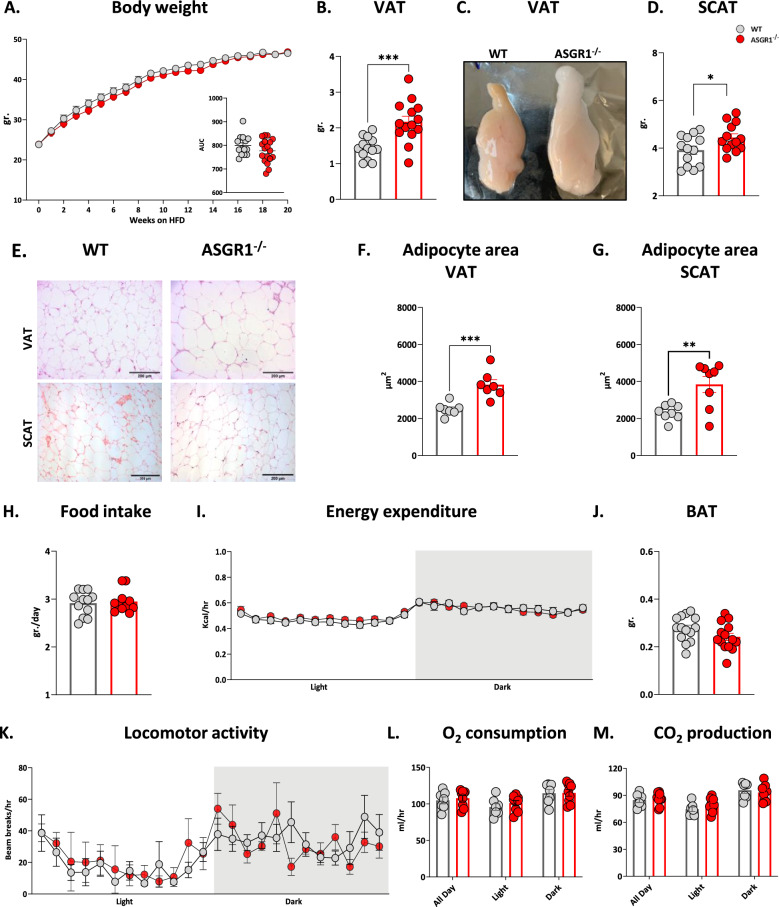
ASGR1 deficiency promotes white adipose tissue hypertrophy on HFD. **A** Body weight over 20 weeks of HFD, in lower right the area under the curve (AUC) for body weight gain calculated over the 20 weeks of HFD of WT and *ASGR1*^*−/−*^ mice. **B** Visceral adipose tissue (VAT) weight after 20 weeks of HFD and (***C***) Representative pictures of VAT from WT and *ASGR1*^*−/−*^ mice. **D** Subcutaneous adipose tissue (SCAT) weight after 20 weeks of HFD in WT and *ASGR1*^*−/−*^ mice. **E** Representative 10 × images of VAT and SCAT sections stained with H&E in WT and *ASGR1*^*−/−*^ mice. **F** Adipocytes area quantification in VAT and **G** SCAT after 20 weeks of HFD in WT and *ASGR1*^*−/−*^ mice. **H** Food intake (grams/day) in WT and *ASGR1*^*−/−*^ mice on HFD. **I** Energy expenditure in WT and *ASGR1*^*−/−*^ mice on HFD measured in metabolic cages as (kcal/hours). *J* Brown adipose tissue (BAT) weight. **K** Locomotor activity **L** O_2_ consumption and **M** CO_2_ production measured using metabolic cages in WT and *ASGR1*^*−/−*^ mice on HFD. Data are obtained from three independent experimental groups. Each bar and error represent the mean ± SEM, *p < 0.05, **p < 0.01 and ***p < 0.001

When fed on HFD, body weight gain in *ASGR1*^−/−^ mice was similar to that of WT mice (Fig. 1A). Nevertheless *ASGR1* ^−/−^ mice presented a 41% increase in visceral adipose tissue (VAT) compared to WT mice (Fig. 1B–C WT 1.54 ± 0.5g., *ASGR1*^−/−^ 2.17 ± 0,6g, p-value < 0.005). Also, the amount of subcutaneous adipose tissue (SCAT) was significantly increased in *ASGR1*^−/−^ compared to WT mice (WT 3.85 ± 0.6g, *ASGR1*^−/−^ 4.42 ± 0.6g, p-value = 0.02) (Fig. 1D). Furthermore, H&E staining of adipose tissue showed more hypertrophic adipocytes in both VAT and SCAT of *ASGR1*^−/−^ mice compared to WT mice (Fig. 1E). The mean of adipocyte area in VAT was 3824.6 ± 719.4 µm^2^ in *ASGR1*^−/−^ and 2495.6 ± 339.4 µm^2^ in WT mice (p < 0.001) (Fig. 1F), while in SCAT was 3843.8 ± 1232.0 µm^2^ in *ASGR1*^−/−^ and 2335.6 ± 412.1 µm^2^ in WT (p-value < 0.01) (Fig. 1G). Notably, these differences were neither the consequence of increased food intake nor of a decreased energy expenditure or of interscapular brown adipose tissue (BAT) accumulation, as these parameters were all comparable between the two experimental groups (Fig. 1H–J). Also, the O_2_ consumption and the CO_2_ production rates, calculated from parameters collected in metabolic cages, were comparable between the experimental groups (Fig. 1K–M). These findings also ruled out the impact of altered non-shivering thermogenesis on the increased adiposity observed in *ASGR1*^−/−^ mice*.*

### ASGR1 deficiency does not alter glucose homeostasis but reduces plasma lipid levels.

**Fig. 2:**
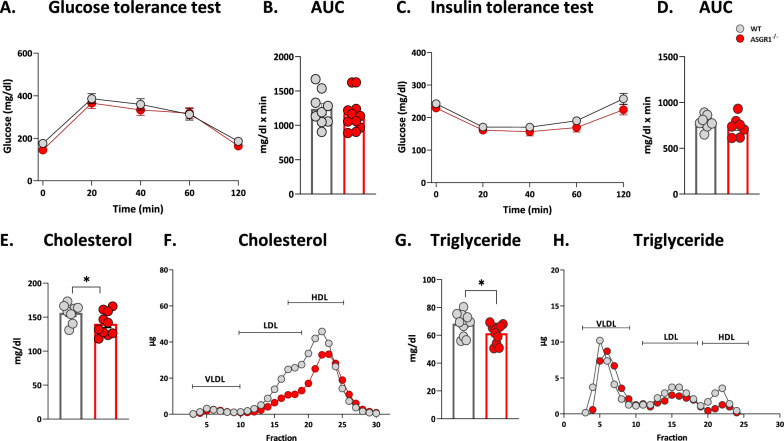
ASGR1 deficiency does not alter glucose homeostasis but reduces plasma lipid levels. **A** Glucose tolerance test (GTT) **B** Area under the curve (AUC) in male wildtype (WT) and *ASGR1*^*−/−*^ mice after 20 weeks of HFD. **C** Insulin tolerance test (ITT) **D** and related AUC in male wildtype (WT) and *ASGR1*^*−/−*^ mice after 20 weeks of HFD. **E** Total plasma cholesterol in male WT and *ASGR1*^*−/−*^ mice after 20 weeks of HFD. **F** Cholesterol content in plasma lipoprotein fractions of male WT and *ASGR1*^*−/−*^ mice after 20 weeks of HFD. **G** Triglycerides concentration in the plasma of male WT and *ASGR1*^*−/−*^ mice after 20 weeks of HFD. **H** Triglyceride content in plasma lipoprotein fractions of male WT and *ASGR1*^*−/−*^ mice after 20 weeks of HFD. Data are obtained from three independent experimental groups. Each bar and error represent the mean ± SEM. *p < 0.05, **p < 0.01 and ***p < 0.001

Subsequently, we assessed whether the increased accumulation of fat in adipose tissue in *ASGR1*^−/−^ mice could be the consequence of impaired glucose metabolism and/or insulin sensitivity. Basal glucose levels were similar in *ASGR1*^−/−^ and WT mice after both 4h and an overnight fasting (Additional file 1: Fig. S2A, B). Moreover, plasma glucose profile was comparable between the study groups following glucose tolerance test (GTT) and insulin tolerance test (ITT) (Fig. 2A–D). By contrast, cholesterol and TG levels in plasma were significantly reduced in *ASGR1*^−/−^ mice compared to WT (Fig. 2E–G) and when we profiled their distribution in the different lipoprotein fractions (by FPLC), we observed a reduction in cholesterol content both in LDL and HDL fractions (Fig. 2F). Instead, TG content was mainly reduced in the HDL fraction (Fig. 2H). To investigate whether the improved circulating lipid profile in *ASGR1*^*−/−*^ mice, could be the consequence of a different fat absorption and/or triglycerides-rich lipoproteins production, we first measured post-prandial elevation of triglycerides after an oral lipid tolerance test. Postprandial triglycerides level elevation was similar in A*SGR1*^−/−^ and in WT animals (Additional file 1: Fig. S2C) and the same was true after intraperitoneal administration of poloxamer, which, by inhibiting triglyceride-rich lipoprotein catabolism, allows to track lipoprotein production during the postprandial phase (Additional file 1: Fig. S2D). These findings indicate that adipose tissue hypertrophy of in *ASGR1*^−/−^ mice is unlikely the consequence of impaired glucose metabolism or lipoprotein production.

### Visceral adipose tissue and liver in ASGR1 deficient mice present a peculiar immunometabolic signature.

**Fig. 3:**
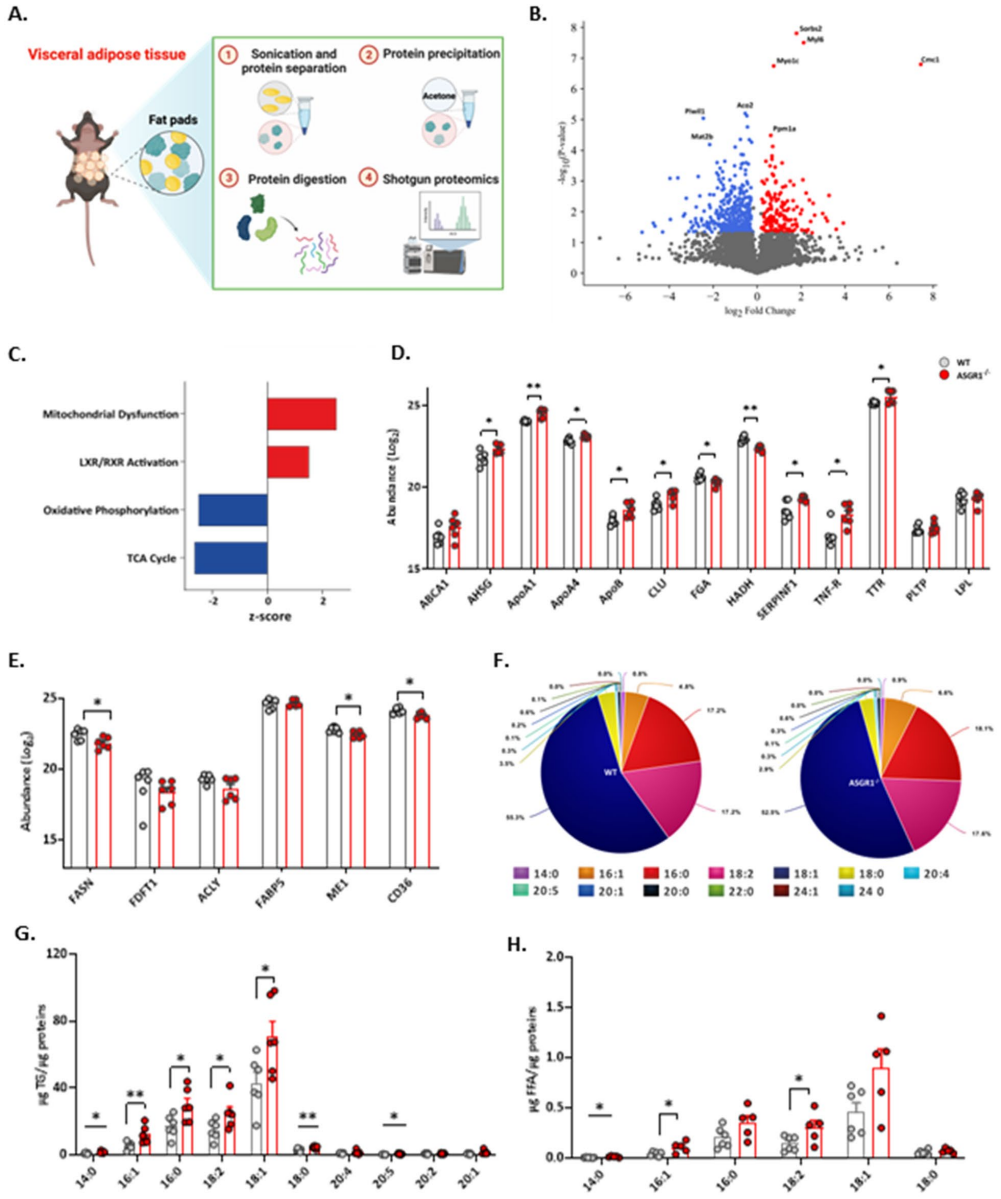
Visceral adipose tissue presents increased lipid content in ASGR1 deficient mice. **A** Scheme depicting VAT processing for shotgun proteomics. Created with Biorender.com. **B** Volcano plot for the differently expressed proteins on VAT of WT and *ASGR1*^*−/−*^ male mice after 20 weeks of HFD. Blue and red represent the down- and upregulated proteins respectively in *ASGR1*^*−/−*^ mice. **C** Canonical pathways for the differently expressed proteins in VAT of WT and *ASGR1*^*−/−*^ male mice after 20 weeks of HFD**.** D–E Abundance (Log_2_-transformed) of the proteins associated with the LXR/RXR pathway. **D** SREBP pathway activation **E** in WT and *ASGR1*^*−/−*^ VAT after 20 weeks of HFD. **F–H** Fatty acid content from the lipid extraction in WT and *ASGR1*^*−/−*^ mice in VAT after 20 weeks of HFD. **F** Total fatty acid distribution **G** Fatty acid content in triglycerides and **H** Free fatty acids (FFA) in VAT of WT and *ASGR1*^−/−^ animals. Each bar and error represent the mean ± SEM. *p < 0.05, **p < 0.01 and ***p < 0.001

The finding described above prompted us to investigate whether VAT hypertrophy could be the consequence of changes in adipocyte storing function. To this aim, we profiled the VAT proteome (Fig. 3A). Overall, the PCA analysis of n = 4196 quantified proteins showed two distinguished proteome signatures between *ASGR1*^−/−^ and WT mice (Additional file 1: Fig. S3A), from which n = 532 exhibited differential expression, where n = 358 showed significant downregulation while n = 174 were significantly upregulated (p-value < 0.05, Fig. 3B**, **Additional file 1: Fig. S3B). The analysis of biological context of the differential expressed proteins (n = 532) in VAT of *ASGR1*^*−/−*^ mice, highlighted the presence of mitochondrial dysfunction coupled with an inhibition of oxidative phosphorylation and TCA cycle (Fig. 3C). Notably we observed the activation of LXR/RXR pathway (Fig. 3C), which is known to stimulate the gene expression involved in lipid uptake and storage [37] also in adipose tissue. The activation of LXR was mirrored in the increase of protein expression of some direct and indirect targets, including α2-HS glycoprotein (AHSG) [38], Apolipoprotein A1 (ApoA1) [39], Apolipoprotein A4 (ApoA4) [40], Apolipoprotein B (ApoB) [38], Clusterin or Apolipoprotein J (CLU) [41], Fibrinogen (FGA) [42], Pigment Epithelium-Derived Factor or Serpin Family F member 1 (SERPINAF1) [43], Tumor necrosis factor receptor superfamily member 1A (TNFR) [44], and transthyretin (TTR) [45] (Fig. 3D). While the activation of LXR induces fatty acid synthesis by controlling SREBP-1c, we observed a significant reduction in the protein levels of specific downstream SREBP1c targets in *ASGR1*^−/−^ compared with the WT mice. This includes fatty acid synthase (FASN), malate dehydrogenase (Me1) and CD36 receptor (CD36) (Fig. 3E). Additionally, in line with the protein expression, the mRNA expression of SREBP1c downstream genes, FASN, acetyl-CoA carboxylase (ACC), stearoyl-Coenzyme A desaturase 1 (SCD1) was significantly decrease in *ASGR1*^−/−^ compared with the WT mice (Additional file 1: Figs. S3C, D).

These findings suggest that in VAT of *ASGR1*^*−/−*^ mice proteins involved in lipogenesis are downregulated while those associated with lipoprotein uptake are increased. Of note, we also observed a significant decrease in proteins related to fatty acid oxidation, palmitic acid modification and cell survival (z-score < -2, Additional file 1: Fig. S3E) and a significant increase in pathways related to lipid accumulation, cholesterol content and activation of antigen presenting cells in *ASGR1*^−/−^ vs WT mice (all with Z-score > 2, Additional file 1: Fig. S3F). These findings suggest that the enhanced lipid availability in VAT of *ASGR1*^−/−^ mice is not appropriately balanced with effective catabolism. To provide insights into lipid composition of VAT we performed lipid extraction and measured the fatty acid content in VAT where the majority belonged to oleic acid (C18:1) followed by linoleic acid (C18:2) and palmitic acid (C16:0) (Fig. 3F). We found a significant increase in the storage of saturated fatty acids (SFAs) such as palmitic acid (C16:0) and stearic acid (C18:0), monounsaturated fatty acids (MUFAs) such as oleic acid (C18:1), and polyunsaturated fatty acids (PUFAs) such as linoleic acid (C18:2) in *ASGR1*^−/−^ mice compared to WT mice (Fig. 3G). Furthermore, the increased lipogenesis index (Additional file 1: Fig. S3G) shows that the VAT of *ASGR1*^−/−^ mice accumulate more lipids than WT animals. Additionally, we observed an increase in free fatty acids (FFA) in VAT of *ASGR1*^−/−^ mice along with an increase in saturated myristic acid (C14:0) and monounsaturated fatty acids such palmitoleic acid (C16:1) and oleic acid (C18:1), suggesting an increase rate of lipolysis and mobilization of accumulated triglycerides in VAT (Fig. 3H). Moreover, we noticed an increasing trend also in the cholesterol ester to free cholesterol ratio in *ASGR1*^−/−^ compared to the WT mice (Additional file 1: Figs. S3H), implying cholesterol homeostasis to be implicated.

**Fig. 4:**
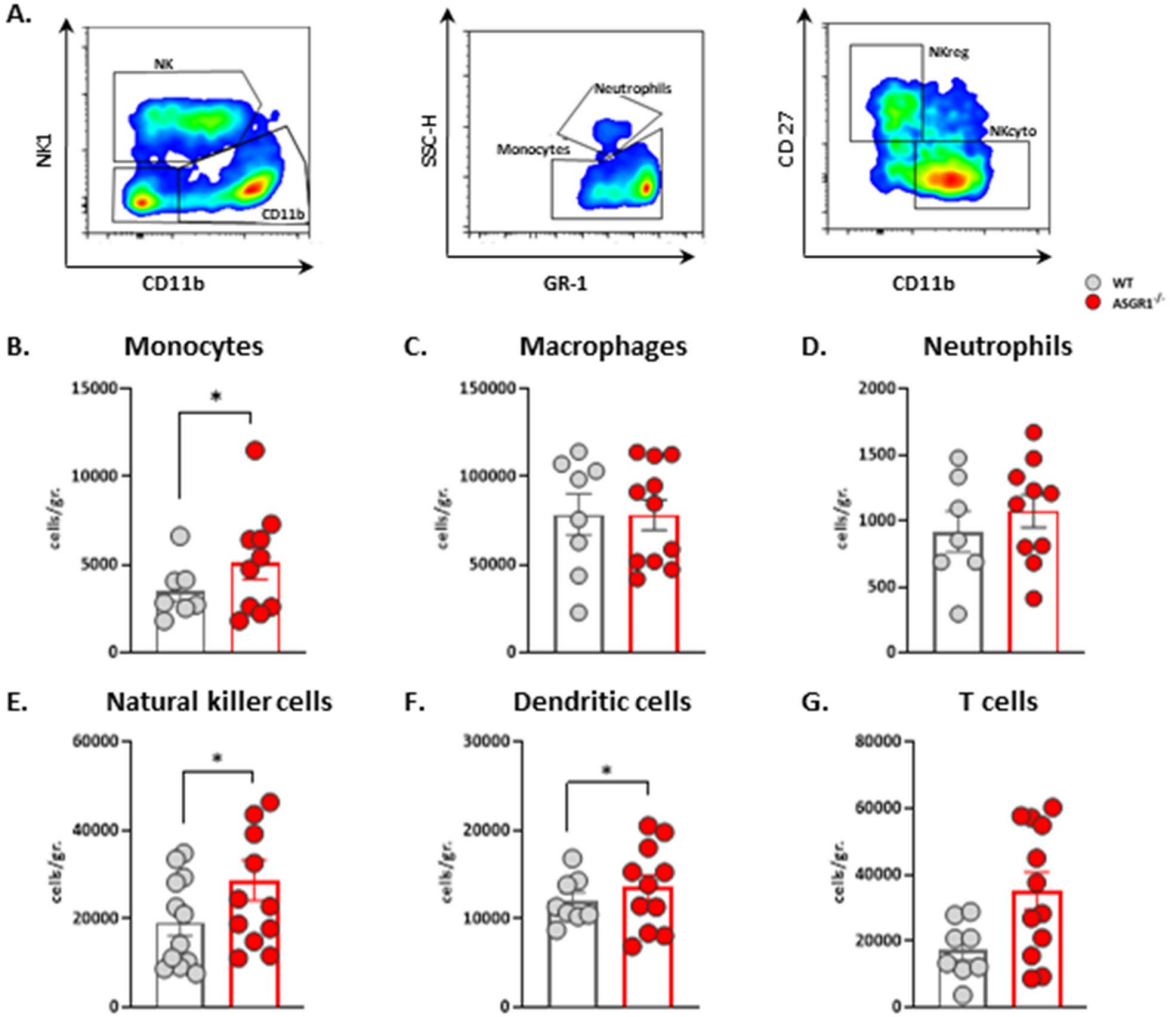
ASGR1 deficiency activates the immune cell surveillance in VAT. VAT immunophenotyping in two experimental groups (n = 8–12), after 20 weeks of HFD. **A** A representative flow cytometry gating strategy for the natural killer cells (NK cells from CD45^+^) and NK subsets (NKreg and NKcito), monocytes and neutrophils. **B–G** Immune cells quantification in WT and *ASGR1*^*−/−*^ VAT after 20 weeks of HFD expressed as cells per gram of tissue. **B** Count in monocytes (GR-1^+^), **C** macrophages (F4/80^+^), **D** neutrophils (SSC-H^high^GR-1^+^), **E** natural killer cells (NK, NK1^+^), **F** dendritic cells (DC, CD 11c^+^) and **G** T cells (CD19^−^ CD3^+^) corrected for the VAT weight (gr.). Each bar and error represent the mean ± SEM. *p < 0.05, **p < 0.01 and ***p < 0.001

Next, we asked whether adipocyte hypertrophy affects immune cell profile and performed an extensive characterization of both innate and adaptive immune cell distribution in VAT (the gating strategy and markers used are described in Fig. 4A**, **Additional file 1: Figs. S4A and S5A). We observed a significant increase of monocytes in VAT of *ASGR1*^−/−^ compared to WT mice (Fig. 4B). Conversely, the levels of macrophages and neutrophils were comparable between both groups (Fig. 4C**, **Fig. 4D). Moreover, VAT in *ASGR1*^−/−^ mice exhibited a higher accumulation of other innate cells, including natural killer (NK, Fig. 4E) and dendritic cells (DCs, Fig. 4F), compared to the WT counterparts. No significant differences in the distribution of cells of the adaptive immune response, including CD3^+^ (T cells, Fig. 4G), CD4 + , CD8 + and B cells (Additional file 1: Fig. S6A–C), were observed.

**Fig. 5:**
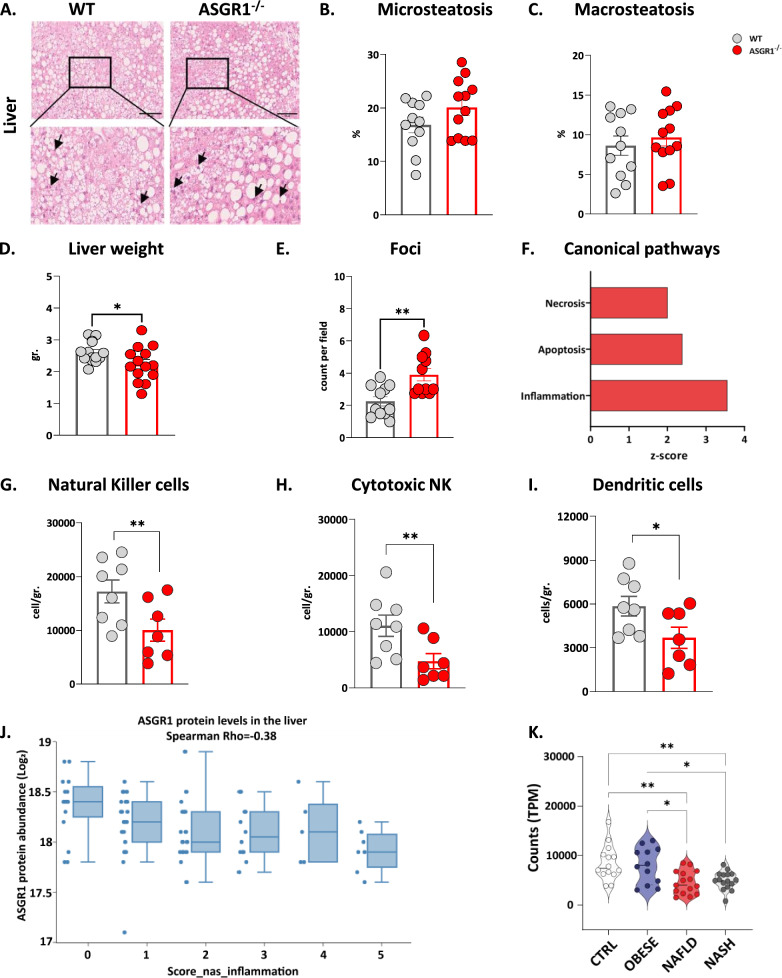
ASGR1 deficiency enhances the risk for liver inflammation. **A** Representative 20 × images of H&E staining of WT and *ASGR1*^*−/−*^ mice livers after 20 weeks of HFD. Percentage of microsteatosis **B** and macrosteatosis **C** in the liver of WT and *ASGR1*^*−/−*^ mice after 20 weeks of HFD. **D** Liver weight after 20 weeks of HFD. **E** Number of inflammatory foci per field in liver sections of WT and *ASGR1*^*−/−*^ mice after 20 weeks of HFD. **F** Activated canonical pathways (z-score > 2) from the differently expressed proteins in the liver. *(G-I)* Flow cytometer analysis of immune cells in the liver of WT and *ASGR1*^*−/−*^ mice after 20 weeks of HFD expressed number of cells/gr of **G** natural killer (NK, NK1^+^)***, H*** cytotoxic NK cells (CD27^+^ CD11b^−^) and **I** dendritic cells (DC, CD 11c^+^)***. (J)*** ASGR1 liver protein abundance (Log_2_-transformed) in patients with different degrees of inflammation (Score_nas_inflammation). **K** Liver ASGR1 gene expression in patients with obesity, NAFLD and NASH. Each bar and error represent the mean ± SEM

Next, we wondered if lipid accumulation could be different also in the liver in ASGR1-deficient conditions. Histological analysis indicated that the presence of microsteatosis and macrosteatosis was similar between WT and *ASGR1*^−/−^ mice (Fig. 5A–C, accordingly with the liver cholesterol and triglyceride content (Additional file 1: Fig. S7A, B). Notably, liver weight was significantly reduced in *ASGR1*^−/−^ mice (Fig. 5D). An in-depth analysis showed an increased number of foci per area in *ASGR1*^−/−^ mice, casting for increased lobular inflamed lesions in this group, compared to WT mice (Fig. 5E). The liver proteome profiling confirmed this hypothesis, as necrosis, apoptosis, and inflammation were among the significantly activated pathways in *ASGR1*^−/−^ compared to WT mice (z-score > 2, Fig. 5F). The analysis of immune cell subsets within the liver revealed a significant reduction in the number of natural killer cells (NK, Fig. 5G), mainly in cytotoxic NK (Fig. 5H, CD11b^+^, CD27^−^) and in dendritic cells (DCs, Fig. 5I) in the liver of *ASGR1*^−/−^ mice compared with the WT while the number of monocytes, macrophages and neutrophiles and CD4^+^ and CD8^+^ cells (Additional file 1: Figs. S6D and S5H respectively) were similar between groups.

Concordantly, we examined human liver datasets encompassing sample previously described for the liver biomarkers for alcohol-related liver disease to assess the correlation between lobular inflammation and the protein expression of the ASGR1 [46]. The analysis revealed a negative correlation (Spearman r = -0.38), indicating that as liver inflammation increases, the protein expression of ASGR1 decreases (Fig. 5J). Additionally, findings from RNA-sequencing data (GSE126848) [47] suggest significant reduction in ASGR1 gene expression as the liver progresses from an obese state to NAFLD and NASH (Fig. 5K).

**Fig. 6:**
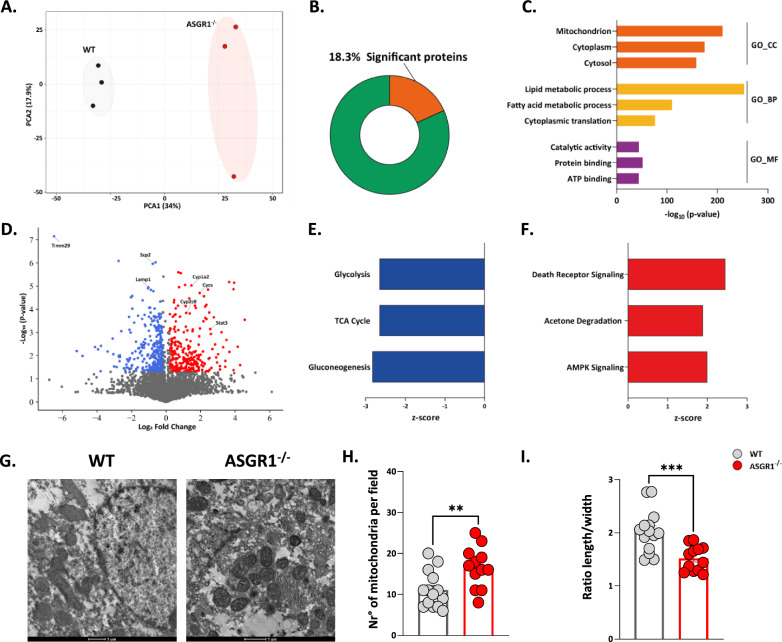
Liver of ASGR1 deficient mice undergoes metabolic rewiring during HFD. **A** Principal component analysis (PCA) of the liver proteome of WT and *ASGR1*^*−/−*^ mice after 20 weeks of HFD. **B** Differently expressed proteins on the total liver proteome of WT and *ASGR1*^*−/−*^ mice after 20 weeks of HFD. **C** Top enriched pathways in gene ontology (GO, FDR < 0.05) for cellular component (CC), biological processes (BP) and molecular function (MF) in the liver proteome of WT and *ASGR1*^*−/−*^ mice after 20 weeks of HFD. **D** Volcano plot for the differently expressed proteins in the liver of WT and *ASGR1*^*−/−*^ mice after 20 weeks of HFD. Depicted blue and red represent the down- and upregulated proteins in *ASGR1*^*−/−*^ mice, respectively. **E**, **F** Metabolic signaling pathways of differently expressed proteins, specifically **E** inhibited (z-score < -2) and **F** activated canonical pathways (z-score > 2) in the liver of *ASGR1*^*−/−*^ mice after 20 weeks of HFD. **G** Representative images of liver photomicrographs obtained by Transmission Electron Microscopy showing mitochondria ultrastructure. Quantification of mitochondria number per field **H** and length to width ratio **I** in WT and *ASGR1*^*−/−*^ mice liver after 20 weeks of HFD. Each bar and error represent the mean ± SEM. *p < 0.05, **p < 0.01 and ***p < 0.001

To further investigate potential changes in the inflammatory and metabolic signature of the liver in ASGR1 deficient conditions, we performed liver proteomics which revealed a distinctive profile in *ASGR1*^−/−^ compared to WT mice (Fig. 6A). Among the 3411 proteins that were identified, 18.3% were differentially expressed between the two groups (p < 0.05, Fig. 6B) with those located in the mitochondria, the cytoplasm, or the cytosol being the prevalent ones (Fig. 6C). A more detailed analysis revealed major changes in biological(BP) encompassing lipid and fatty acid metabolism and cytoplasmic translation (Fig. 6C), and pathways related to catalytic activity, protein binding and ATP binding (Fig. 6C). The most increased proteins, depicted in red in volcano plot, belong to cytochrome P450 oxidases such as CYP1A2, CYP2C8, CYP2E1 and the transcription factor STAT3 (Fig. 6D), which are known to be activated during processes associated to liver damage.

By contrast, among the most significantly downregulated proteins (in blue), we observed lysosomal-associated membrane protein (LAMP), which is critical in autophagy and in immune response by presenting the antigens on the cell surface, and the sterol carrier protein 2 (*SCP2*), which plays a role in the cellular lipid storage. Furthermore, the analysis of canonical pathways revealed that the liver of *ASGR1*^*−/−*^ mice presents the inhibition of glycolysis, tricarboxylic acid cycle (TCA), and gluconeogenesis (z-score < 2, Fig. 6E), along with activation of acetone degradation and AMPK signaling and cell death signaling (z-score > 2, **Fig. F**) suggestive of a condition of liver damage. Indeed, electron transmission microscopy (TEM, Fig. 6G) of liver sections revealed that *ASGR1*^−/−^ mice display swelling and a disorganization of mitochondrial cristae and mitochondria-associated ER membranes. In *ASGR1*^−/−^ mice, mitochondria were spherical in shape, as opposed to the elongated mitochondria of WT mice. Indeed, the number of mitochondria per area was significantly higher in *ASGR1*^−/−^ compared with the WT mice (WT 11.1% ± 1.2, *ASGR1*^−/−^ 16.6% ± 1.4, p-value < 0.01, Fig. 6H). Accordingly, we observed an increased length/width ratio (Fig. 6I), which is indicative of mitochondrial dysfunction. Additionally, we focused on proteins implicated in oxidative phosphorylation (FDR < 0.01) and comprehensive proteomic heatmap divided by mitochondrial complex I (Additional file 1: Fig. S7C), complex II, III, IV (Additional file 1: Fig. S7D), and complex V (Additional file 1: Fig. S7E) here a significant decrease is appreciated in the ATP-production of the complex V. These findings indicate that the liver in *ASGR1*^−/−^ mice under HFD feeding becomes dysfunctional.

## Discussion

Our study demonstrates that ASGR1 plays a critical role in maintaining metabolic flexibility required during excessive caloric intake seen under a chronic exposure of HFD. The metabolic consequences of the absence of ASGR1, resulted in a shift of the excess fat, which would normally be processed in the liver, towards adipose tissue. As a result, fat accumulating in visceral adipose tissue in ASGR1 deficient mice increased remarkably by 41% when comparing to the WT. Noteworthy, adipose tissue serves as the major site for lipid uptake, thus, the fat directed towards adipose tissue prevents metabolic syndrome [48]. Additionally, despite the adipocytes hypertrophy, *ASGR1*^*−/−*^ mice maintained a comparable glucose and insulin response during an acute challenge compared to the WT indicating for an overall functionality of their systemic metabolism. This is however accompanied by a deteriorated liver function.

Consistent with these findings, we noted a decrease in plasma cholesterol and TG levels in *ASGR1*^*−/−*^ mice and this reduction was evident in both LDL and HDL fraction for cholesterol, whereas for TG we observed a primarily decrease in the HDL fraction. It is worth noting that as reported cholesterol accumulation within lipid droplets of adipocytes is proportional to TG content [49, 50], and it is coupled with a decrease in plasma cholesterol levels in hypertrophic adipocytes [51]. The analysis of the VAT proteome showed that the LXR/RXR pathway, which is known to regulate the expression of several target genes involved in adipose tissue lipid storage and adipogenesis [52], appears to be more activated in VAT from *ASGR1*^*−/−*^ mice compared to WT mice. Several studies, however, indicated that the regulation of lipogenesis in adipocytes differs from that in the liver and that although LXR induced SREBP-1c gene expression in VAT this was not matched with an increased expression of *FASN* [53, 54]. This was also our case, where FASN expression was reduced in VAT from *ASGR1*^−/−^ mice compared to WT. We might speculate that the excessive cholesterol accumulation in VAT activates the LXR pathway to favor its elimination but, at the same time, as adipocytes already store an enormous amount of TG, it should inhibit lipogenesis. Moreover, in VAT we found a significant increase in the storage of SFAs, MUFAs as well as PUFAs in *ASGR1*^−/−^ mice compared to WT mice. In parallel the FFA in VAT of *ASGR1*^−/−^ mice were increased, suggesting an increase rate of lipolysis to mobilize the accumulation of TG in VAT. It remains to be explored how only a part of LXR-driven protein expression is activated and a mechanistic cell-culture approach could elucidate the potential mechanisms of lipid accumulation in adipocytes in ASGR1 deficiency. The observation of a non-healthy hypertrophic adipose tissue is coupled to the increased prevalence of certain immune cell subsets including NK, DCs, and monocytes in agreement with other studies with a similar phenotype [55].

Remarkably, *ASGR1*^*−/−*^ mice did not present steatosis nor insulin resistance compared to WT mice, suggesting that the intracellular fat already present in the liver is oxidized rather than accumulated as intracellular fat to induce steatosis. Moreover, *ASGR1*^*−/−*^ mice presented a decrease in TCA cycle, glycolysis as well as gluconeogenesis, being the classical sources of energy, along with the activation of acetone degradation, the final product generated by the hydrolysis of fatty acids, suggesting that livers from ASGR1 deficient mice adjust the metabolic needs by shifting the energy metabolism towards fatty acids, decreasing the reliance on glucose as a fuel source. Additionally, ASGR1^−/−^ mice presented an activation of AMPK, in line with Wang et al. findings, hinting for a selective modulation of AMPK in the ASGR1 deficiency [17]. Since AMPK functions as an energy sensing master regulator, its activation stimulates the mitochondrial biogenesis through enhanced fatty acid oxidation while simultaneously inhibiting fatty acid and cholesterol synthesis [19]. Additionally, STAT3, one of the most upregulated proteins in the liver of *ASGR1*^*−/−*^ mice, enhances electron respiratory chain activity and ATP production by interacting with complexes I and II and consequently increasing NADH [56]. Nevertheless, persistent activation of STAT3 in the liver has been associated with the development and progression of apoptosis and fibrosis in the liver fibrosis [57]. Indeed, the liver from *ASGR1*^−/−^ mice presents increased inflammatory foci and altered hepatocyte mitochondrial structure. This observation, coupled to the reduction of NK and DCs that are actively involved immune surveillance [58], suggests the presence of liver dysfunction.

In human, the expression of ASGR1 was observed to be significantly decreased in patients with NAFLD and NASH as well as in hepatic cirrhosis or in highly proliferative liver tumors [46, 59–61],. Accordingly, our liver histology and proteomics data showed that *ASGR1*^−/−^ mice also presented the activation of apoptosis and necrosis, consistent with the findings observed in ASGR1 deficient pigs [62]. Interestingly, in humans the loss of function in ASGR1 has been associated with elevated levels of alkaline phosphate (ALP) in circulation. However, there were no significant changes in the levels of gamma-glutamyltransferase, bilirubin and alanine aminotransferase, thus, the increased ALP levels in carriers is attributed to the compromised clearance of desialylated proteins in the circulation [16]. Nevertheless, we did not observe any change in N-glycome, including desilaylated glycoproteins, in *ASGR1*^*−/−*^ compared to the WT mice [63], thus, it is possible that the increase in ALP levels might reflect the presence of liver dysfunction.

In summary, despite its beneficial impact on plasma lipid levels, the ASGR1 deficiency results in markers of altered energetic homeostasis during HFD induced obesity, being reflected by increased lipid accumulation in VAT and liver damage.

### Supplementary Information

**Additional file 1: Figure S1. **Weight and energy metabolism. **A** Total body weight after 20 weeks of high-fat diet (HFD). **B** Spleen and **C** Mesenteric lymph nodes (mLN) after 20 weeks of HFD. Each bar and error represent the mean ± SEM. **Figure S2. **Circulating glucose and lipid homeostasis. **A** Basal glucose after 14h of fasting before glucose tolerance test (GTT) in WT and ASGR1^−/−^ mice after 20 weeks of high-fat diet (HFD). **B** Basal glucose after 4h of fasting before insulin tolerance test (ITT) in WT and ASGR1^−/−^ mice after 20 weeks of HFD. **C** Triglycerides levels during the oral fat load and **D** after poloxamer injection in WT and ASGR1^−/−^ mice after 20 weeks of HFD. **E** Plasma aspartate aminotransferase (AST) levels and **F** plasma alanine transaminase (ALT) in WT and ASGR1^−/−^ mice after 20 weeks of HFD. Each bar and error represent the mean ± SEM. **Figure S3. **Visceral adipose tissue metabolism.* A* Principal component analysis (PCA) of the VAT proteome of WT and *ASGR1*^*−/−*^ mice after 20 weeks of HFD. **B** Percentage of differently expressed proteins in VAT samples of WT and ASGR1^−/−^ mice after 20 weeks of high-fat diet (HFD). **C**, **D** Gene expression of transcription factors and LXR-targets***. E*** Inhibited metabolic pathways (z-score < -2) and **F**activated metabolic pathways (z-score > 2) for the significantly differentially expressed proteins in WT and *ASGR1*^*−/−*^ VAT after 20 weeks of HFD. **G** Lipogenesis index (C16:0/C18:2), (***H***) CE/FC ratio and (**I**) Phospholipid content (nmol/μg proteins) in WT and *ASGR1*^*−/−*^ VAT after 20 weeks of HFD. Each bar and error represents the mean ± SEM. *p < 0.05, **p < 0.01 and ***p < 0.001. **Figure S4. **Flow cytometry gating strategy for the analysis of innate immune cells. A Doublets were first excluded by the analysis (FSC-H vs. FSC-A). Leukocytes were gated based on dimension (FSC-H vs SSC-H) and CD45 positivity. Immune cells were identified as follows: B cells (CD19^+^, CD3^−^), T cells (CD19^−^,CD3^+^), macrophages (CD19^−^,CD3^−^,F4/80^+^), Natural killer cells (NK, CD19^−^,CD3^−^,F4/80^−^,NK1^+^), monocytes (NK1^−^,CD11b^+^,GR-1^+^ SSC-H^low^ among F4/80^−^ cells), neutrophils (NK1^−^,CD11b^+^,GR-1^+^,SSC-H^high^ among F4/80^−^ cells), regulatory NK cells (CD27^+^,CD11b^−^ among NK1^+^), cytotoxic NK cells (CD27^+^,CD11b^− ^among NK1^+^), dendritic cells (DC, CD11c^+^ among F4/80^−^ cells). **Figure S5**. Flow cytometry gating strategy for the analysis of adaptive immune cells. **A** Cells were gated on dimension (FSC-H vs SSC-H) and single cells discriminated (FSC-H vs. FSC-A). Leukocytes were then identified as CD45^+^ and T cells as CD3^+^ cells (CD45^+^, CD3^+^). Among CD45^+^CD3^+^ cells, CD4^+^ (T helper) and CD8^+^ (T cytotoxic) T cells were selected. **Figure S6. **VAT and liver immunophenotyping. The experiments are performed in three experimental groups (n = 6–14), after 20 weeks of high-fat diet (HFD). Immunophenotypic analysis through flow cytometry of VAT in WT and ASGR1^−/−^ mice after 20 weeks of HFD: cells per gram of tissue of T helper cells (CD4^+^) **A**, cytotoxic T cells (CD8^+^) **B** and B cells (*C*). **D–H** Immunophenotypic analysis through flow cytometry of the liver in WT and ASGR1^−/−^ mice after 20 weeks of HFD:** D** Monocytes **E** Macrophages **F** Neutrophils **G** CD 4^+^ T cells **H** CD 8^+^ T cells expressed as cells per gram of tissue. Each bar and error represent the mean ± SEM. **Figure S7. **Liver lipids and metabolism. (**A**) Liver cholesterol levels and (**B**) Liver triglyceride levels in WT and ASGR1^−/−^ mice after 20 weeks of HFD. (**C**–**E**) Heatmap with Pearson clustering representing the oxidative phosphorylation pathway (FDR < 0.01) subdivided in **C** Mitochondrial complex I (**D**) Mitochondrial complex II, III and IV (**E**) Mitochondrial complex V.

**Additional file 2: Table S1.** List of fluorescent-conjugated antibodies. **Table S2.** The LFQ-abundance of visceral adipose tissue proteome. **Table S3.** The LFQ-abundance of liver proteome. **Table S4.** The sequences of the mouse qPCR primers.

## Data Availability

The mass spectrometry proteomics data have been deposited to the ProteomeXchange Consortium [64] via The PRIDE partner repository with reference number PXD046809.

## Abbreviations

AMPK: AMP-activated protein kinase
ASGPR: Asialoglycoprotein receptor
ASGR1: Asialoglycoprotein receptor 1
ASGR1^−^^/^^−^: Asialoglycoprotein deficient mice
Chol: Cholesterol
GTT: Glucose tolerance test
HDL: High-density lipoprotein
HFD: High-fat diet
ITT: Insulin tolerance test
LDL: Low-density lipoprotein
LFQ: Label-free quantification
LXR: Liver X receptor
SCAT: Subcutaneous adipose tissue
SREBP-1c: Sterol regulatory element-binding protein
TGs: Triglycerides
VAT: Visceral adipose tissue
VLDL: Very-low density lipoprotein
