# Supplementary material for: ASGR1 deficiency diverts lipids toward adipose tissue but results in liver damage during obesity
Source: Cardiovasc Diabetol. 2024 Jan 28;23:42. doi: 10.1186/s12933-023-02099-6 (PMC10823681; doi:10.1186/s12933-023-02099-6)
Supplement: Supplementary file 1 — Additional file 1: Figure S1. Weight and energy metabolism. A Total body weight after 20 weeks of high-fat diet (HFD). B Spleen and C Mesenteric lymph nodes (mLN) after 20 weeks of HFD. Each bar and error represent the mean ± SEM. Figure S2. Circulating glucose and lipid homeostasis. A Basal glucose after 14h of fasting before glucose tolerance test (GTT) in WT and ASGR1−/− mice after 20 weeks of high-fat diet (HFD). B Basal glucose after 4h of fasting before insulin tolerance test (ITT) in WT and ASGR1−/− mice after 20 weeks of HFD. C Triglycerides levels during the oral fat load and D after poloxamer injection in WT and ASGR1−/− mice after 20 weeks of HFD. E Plasma aspartate aminotransferase (AST) levels and F plasma alanine transaminase (ALT) in WT and ASGR1−/− mice after 20 weeks of HFD. Each bar and error represent the mean ± SEM. Figure S3. Visceral adipose tissue metabolism. A Principal component analysis (PCA) of the VAT proteome of WT and ASGR1−/− mice after 20 weeks of HFD. B Percentage of differently expressed proteins in VAT samples of WT and ASGR1−/− mice after 20 weeks of high-fat diet (HFD). C, D Gene expression of transcription factors and LXR-targets. E Inhibited metabolic pathways (z-score < -2) and Factivated metabolic pathways (z-score > 2) for the significantly differentially expressed proteins in WT and ASGR1−/− VAT after 20 weeks of HFD. G Lipogenesis index (C16:0/C18:2), (H) CE/FC ratio and (I) Phospholipid content (nmol/μg proteins) in WT and ASGR1−/− VAT after 20 weeks of HFD. Each bar and error represents the mean ± SEM. *p < 0.05, **p < 0.01 and ***p < 0.001. Figure S4. Flow cytometry gating strategy for the analysis of innate immune cells. A Doublets were first excluded by the analysis (FSC-H vs. FSC-A). Leukocytes were gated based on dimension (FSC-H vs SSC-H) and CD45 positivity. Immune cells were identified as follows: B cells (CD19+, CD3−), T cells (CD19−,CD3+), macrophages (CD19−,CD3−,F4/80+), Natural killer cells (NK, CD19−,CD3−,F4 [file 12933_2023_2099_MOESM1_ESM.pdf]

Supplementary data

ASGR1 deficiency directs lipids towards adipose tissue in obesity.

Monika Svecla[1], Lorenzo Da Dalt[1], Annalisa Moregola[1], Jasmine Nour[1], Andrea Baragetti[1], Patrizia Uboldi[1], Elena Donetti[2], Lorenzo Arnaboldi[1], Giangiacomo Beretta[3], Fabrizia Bonacina[1], Giuseppe Danilo Norata[1]

[1]Department of Pharmacological and Biomolecular Sciences, Università degli Studi di Milano, Milan, Italy.

[2]Department of Biomedical Science for Health, Università degli Studi di Milano, Italy. [3]Department of Environmental Science and Policy, Università degli Studi di Milano, Milan, Italy.

Corresponding author:

Giuseppe Danilo Norata, Professor of Pharmacology, University of Milan,Via Balzaretti 9, 20133 Milan, Italy. E-mail: danilo.norata@unimi.it

Supplementary data

Supplemental content (this file):

Supplementary Figure S1 – Weight and energy metabolism.

Supplementary Figure S2 – Circulating glucose and lipid homeostasis.

Supplementary Figure S3 – Visceral adipose tissue metabolism.

Supplementary Figure S4 – Flow cytometry gating strategy for the analysis of innate immune cells.

Supplementary Figure S5 –Flow cytometry gating strategy for the analysis of adaptive immune cells.

Supplementary Figure S6 – VAT and liver immunophenotyping.

Supplementary Figure S7– Liver lipids and metabolism.

Supplemental content (excel file):

Supplementary Table S1 – List of fluorescent-conjugated antibodies

Supplementary Table S2 – The LFQ-abundance of visceral adipose tissue proteome

Supplementary Table S3 – The LFQ-abundance of liver proteome

Supplementary Table S4 – The sequences of the mouse qPCR primers

Supplementary Figure S1

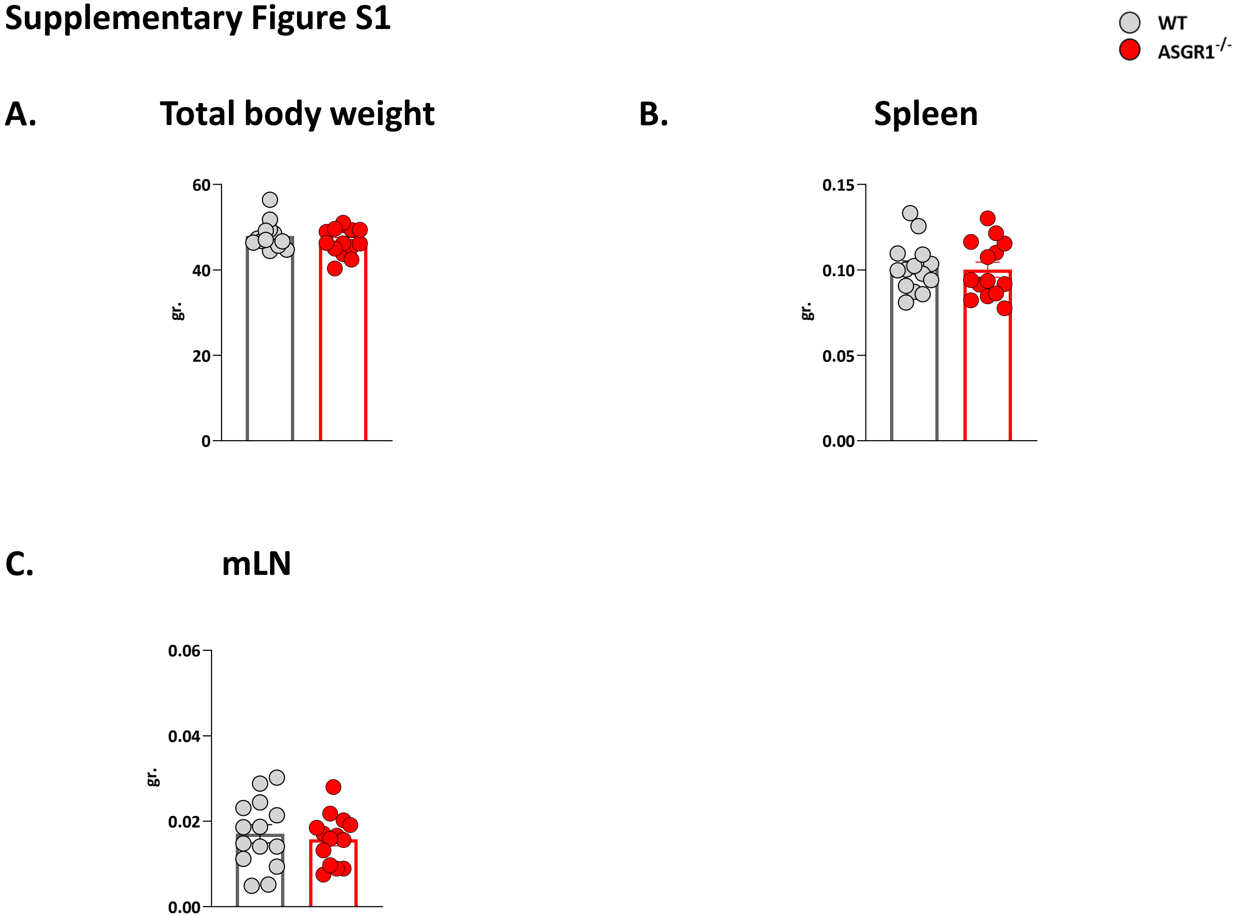

Supplementary Figure S1 – Weight and energy metabolism.

Supplementary Figure S2

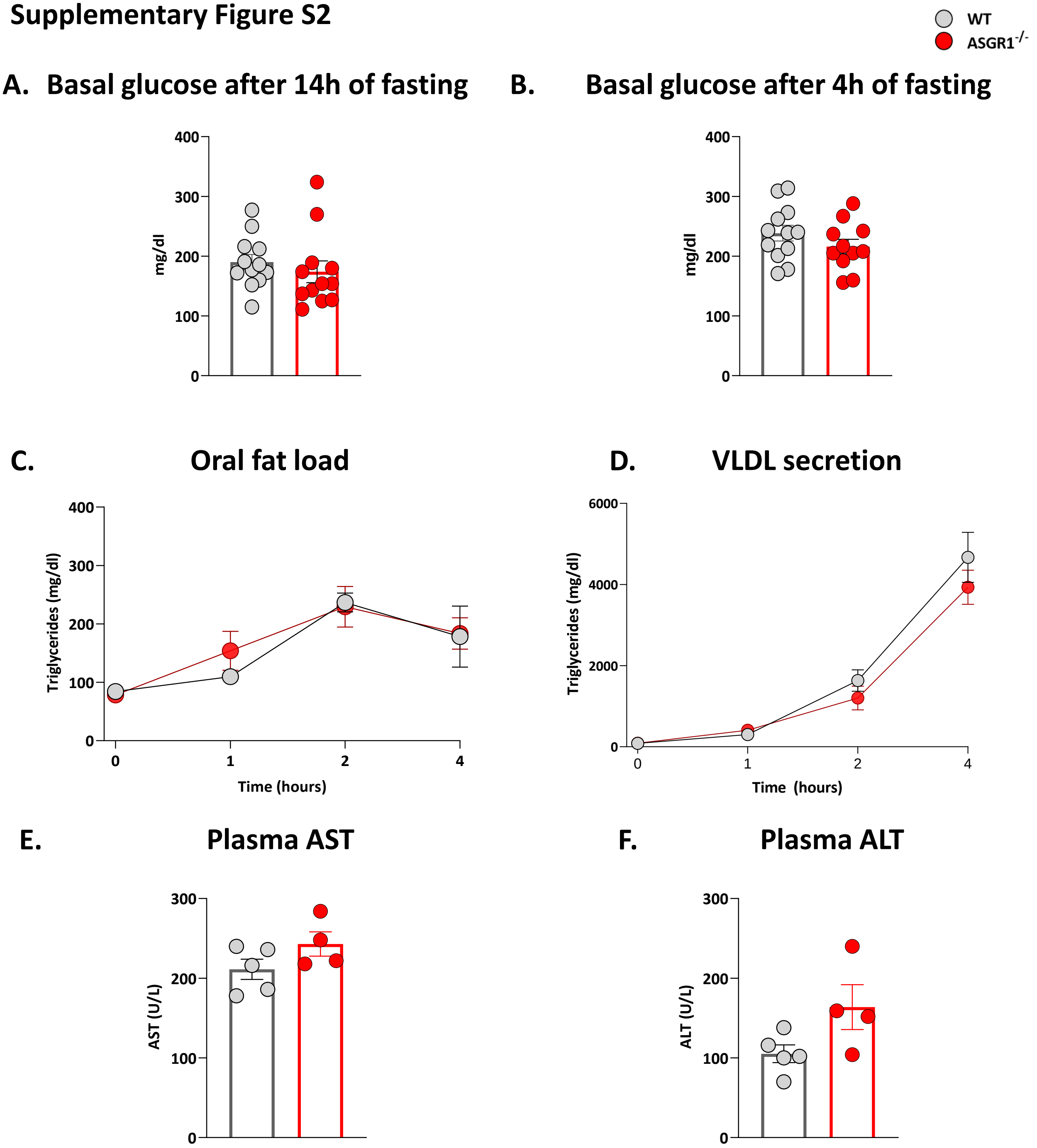

Supplementary Figure S2 – Circulating glucose and lipid homeostasis.

Supplementary Figure S3

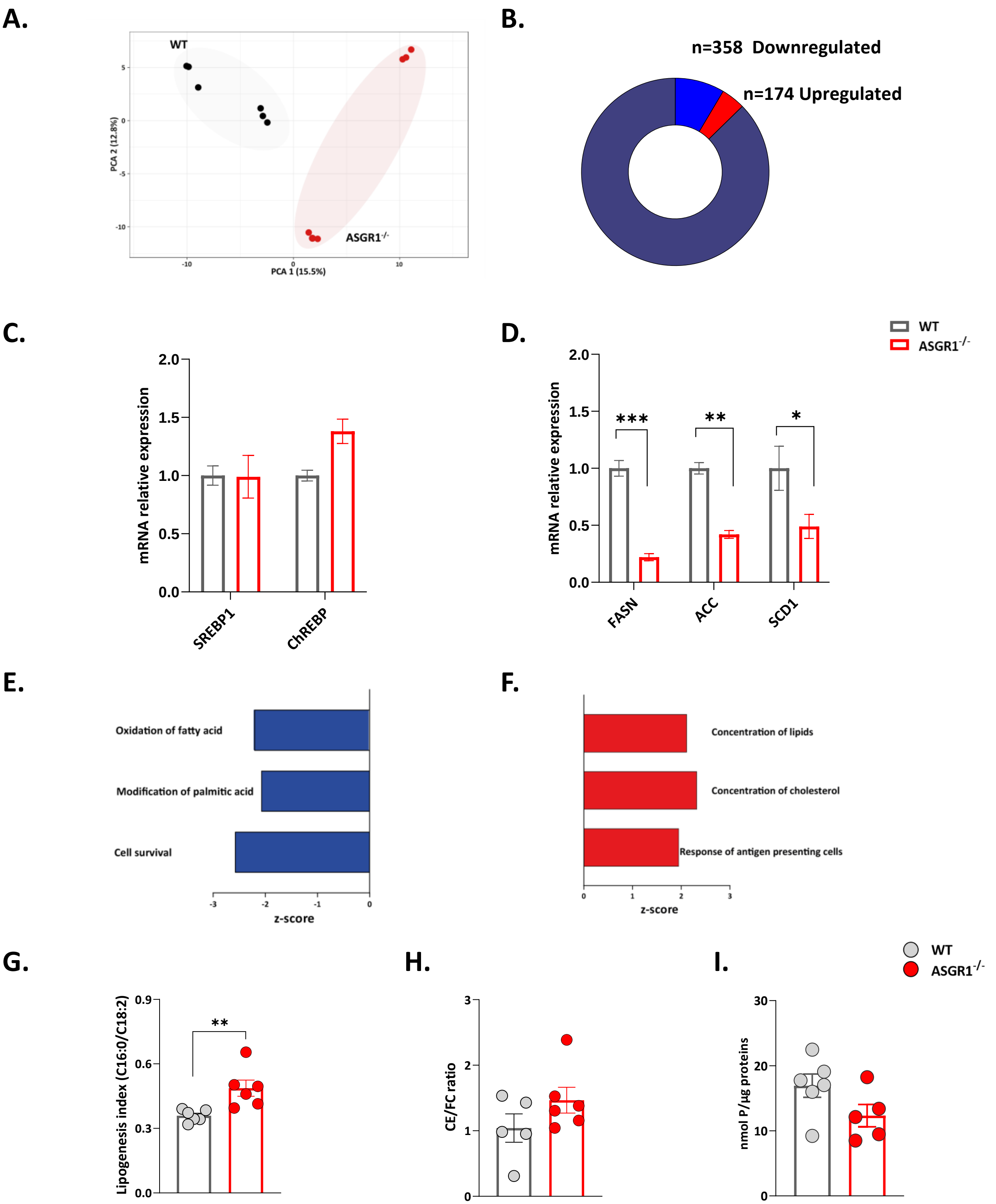

Supplementary Figure S3 – Visceral adipose tissue metabolism

Supplementary Figure S4

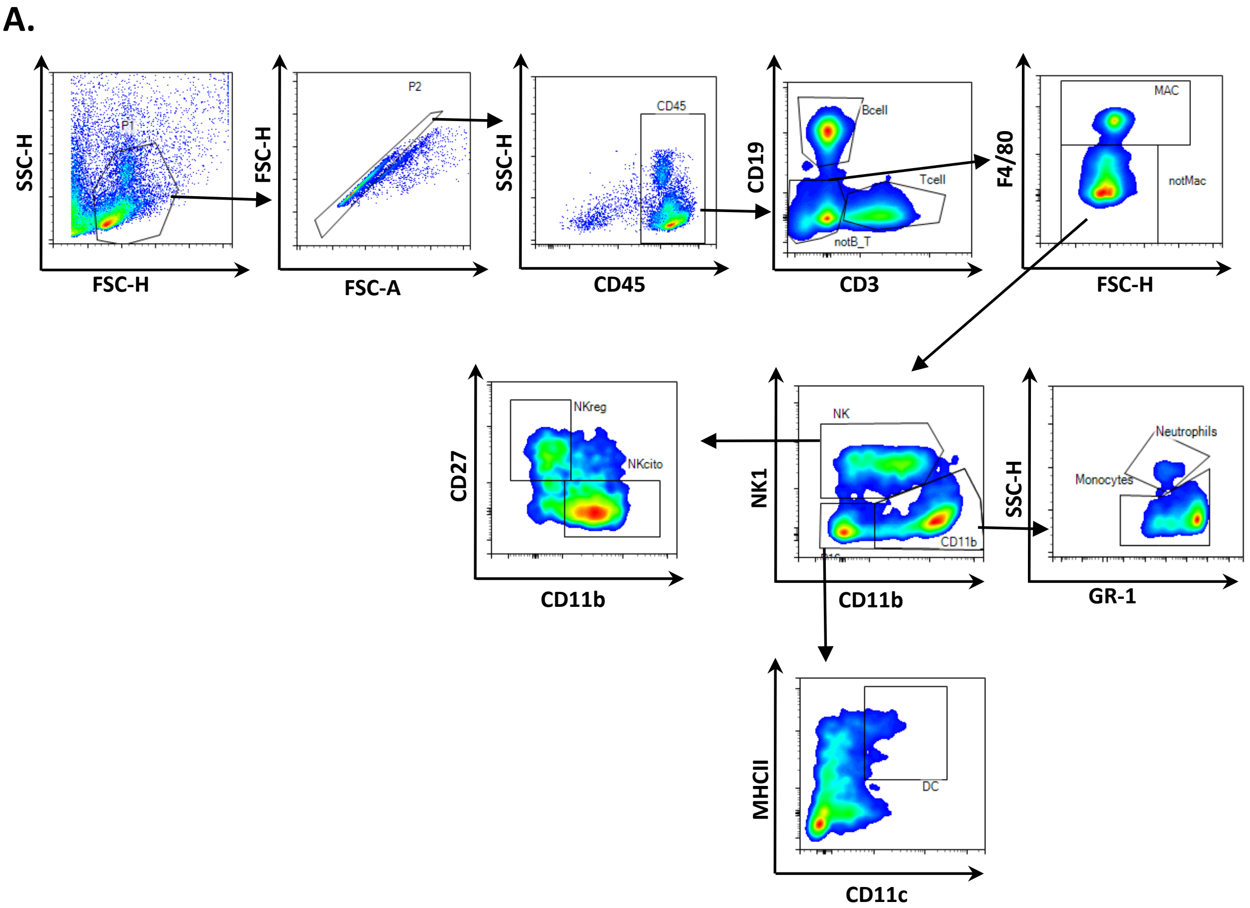

Supplementary Figure S4 –Flow cytometry gating strategy for the analysis of innate immune cells.

Supplementary Figure S5

A.

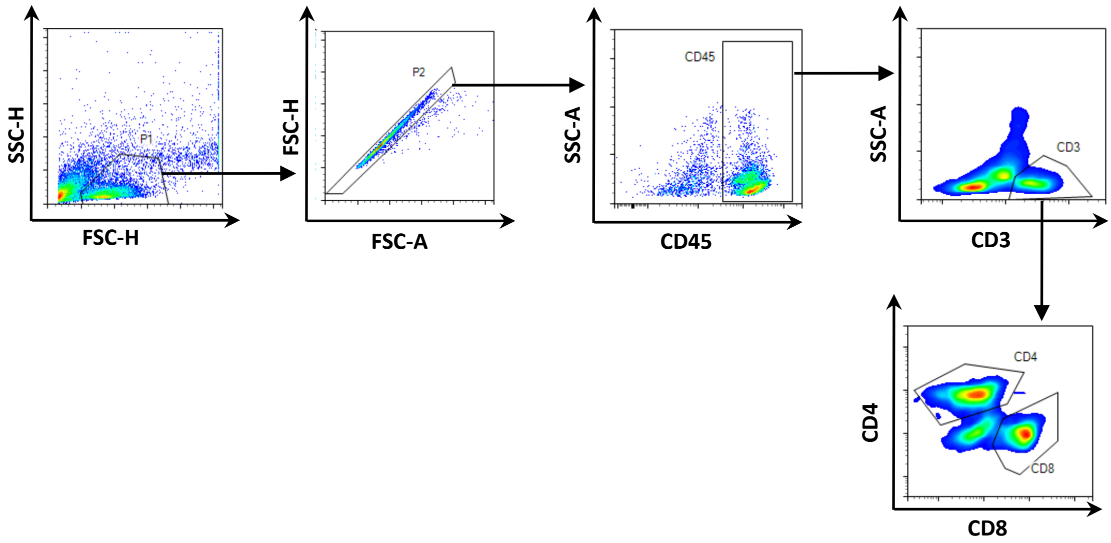

Supplementary Figure S5 – Flow cytometry gating strategy for the analysis of adaptive immune cells.

Supplementary Figure S6

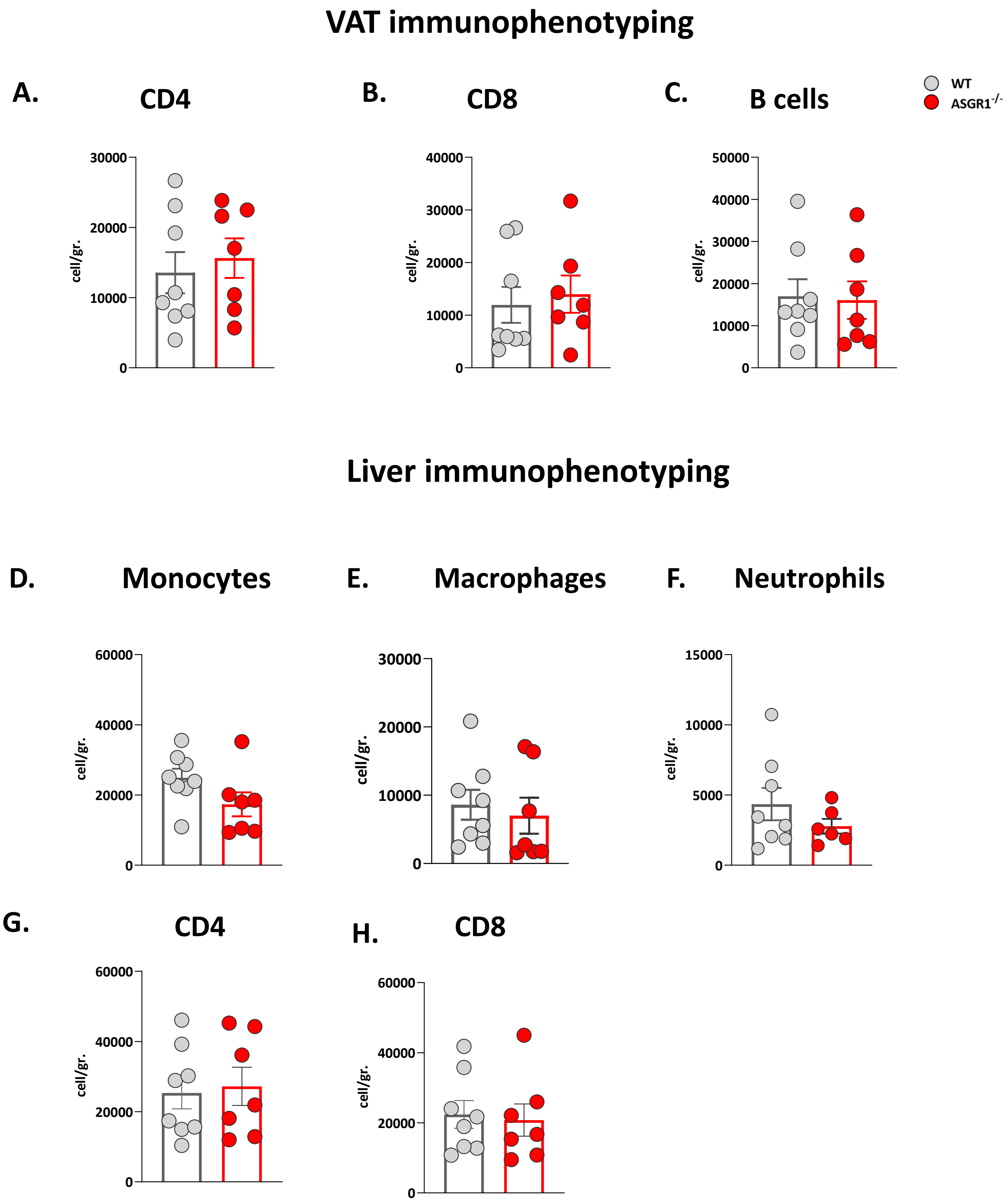

Supplementary Figure S6 – VAT and liver immunophenotyping.

Supplementary Figure S7

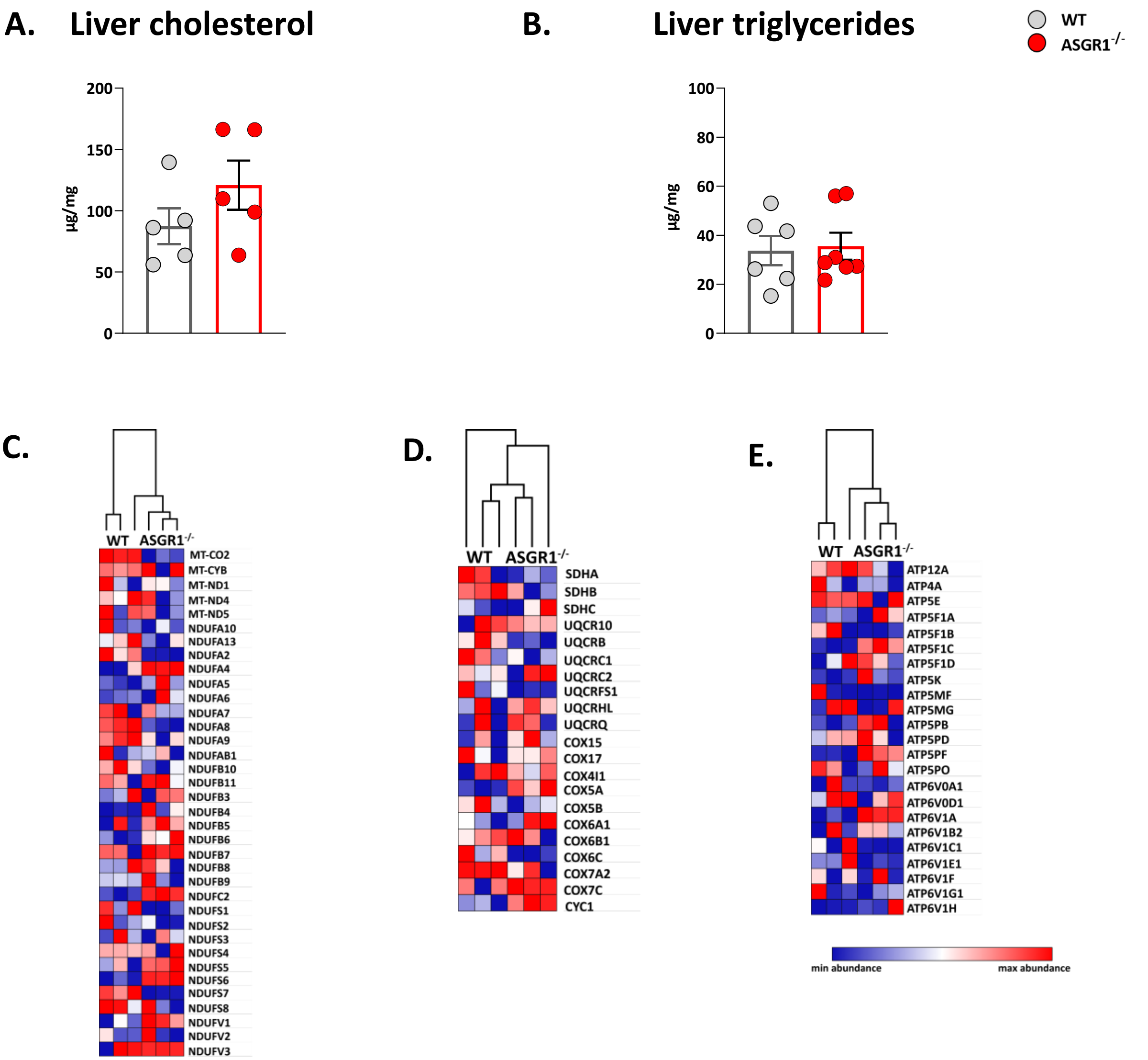

Supplementary Figure S7 – Liver lipids and metabolism
